# UV-B inhibition of hypocotyl growth in etiolated *Arabidopsis thaliana* seedlings is a consequence of cell cycle arrest initiated by photodimer accumulation

**DOI:** 10.1093/jxb/eru035

**Published:** 2014-03-03

**Authors:** Jessica J. Biever, Doug Brinkman, Gary Gardner

**Affiliations:** Department of Horticultural Science, University of Minnesota, St Paul, MN 55108, USA

**Keywords:** *Arabidopsis*, cell cycle arrest, DNA repair, hypocotyl growth, nucleotide excision repair, photodimers, photomorphogenesis, photoreactivation, UV-B.

## Abstract

Ultraviolet (UV) radiation is an important constituent of sunlight that determines plant morphology and growth. It induces photomorphogenic responses but also causes damage to DNA. *Arabidopsis* mutants of the endonucleases that function in nucleotide excision repair, *xpf-3* and *uvr1-1*, showed hypersensitivity to UV-B (280–320nm) in terms of inhibition of hypocotyl growth. SOG1 is a transcription factor that functions in the DNA damage signalling response after γ-irradiation. *xpf* mutants that carry the *sog1-1* mutation showed hypocotyl growth inhibition after UV-B irradiation similar to the wild type. A DNA replication inhibitor, hydroxyurea (HU), also inhibited hypocotyl growth in etiolated seedlings, but *xpf-3* was not hypersensitive to HU. UV-B irradiation induced accumulation of the G_2_/M-specific cell cycle reporter construct *CYCB1;1-GUS* in wild-type *Arabidopsis* seedlings that was consistent with the expected accumulation of photodimers and coincided with the time course of hypocotyl growth inhibition after UV-B treatment. Etiolated mutants of *UVR8*, a recently described UV-B photoreceptor gene, irradiated with UV-B showed inhibition of hypocotyl growth that was not different from that of the wild type, but they lacked UV-B-specific expression of chalcone synthase (*CHS*), as expected from previous reports. *CHS* expression after UV-B irradiation was not different in *xpf-3* compared with the wild type, nor was it altered after HU treatment. These results suggest that hypocotyl growth inhibition by UV-B light in etiolated *Arabidopsis* seedlings, a photomorphogenic response, is dictated by signals originating from UV-B absorption by DNA that lead to cell cycle arrest. This process occurs distinct from *UVR8* and its signalling pathway responsible for *CHS* induction.

## Summary

Inhibition of hypocotyl growth in etiolated *Arabidopsis* seedlings is due to cell cycle arrest initiated through direct absorption of UV-B by DNA and subsequent photodimer accumulation, and does not require UVR8.

## Introduction

Plants have evolved sophisticated systems for perceiving and responding to a wide array of environmental stimuli. Among these is the perception of light signals through photoreceptors that absorb light at specific wavelengths. Ultraviolet (UV) radiation is a particularly important part of sunlight that dictates plant morphology and growth. UV-B light (280–320nm), specifically, is a unique light stimulus in that it induces photomorphogenic responses in plants and also causes damage to biomolecules such as DNA. Many years ago, action spectra of several plant responses to UV irradiation implicated DNA as the main chromophore based on relative photon effectiveness weighted to 280nm (Caldwell, 1971). However, plant responses to UV-B-induced DNA damage are often considered a general reaction to stress rather than a specific consequence of UV-B light perception (Brosché and Strid, 2003; Frohnmeyer and Staiger, 2003).

When DNA absorbs UV-B light, energy from the photon causes covalent linkages to form between adjacent pyrimidine bases, creating photodimers (Taylor, 2006), primarily cyclobutane pyrimidine dimers (CPDs) and pyrimidine-6,4-pyrimidinone dimers (6,4PPs). Photodimers create such distortions in the DNA strand that they block transcription and replication (Britt, 2004). Accumulation of photodimers is harmful to overall plant growth and genome integrity if they are not repaired (Ries *et al.*, 2000), and UV-B photodimers can activate DNA damage response pathways that result in cell cycle arrest or programmed cell death in stem cells of the root apical meristem (Curtis and Hays, 2007; Furukawa *et al.*, 2010). Fortunately, plants have fairly robust mechanisms to repair photodimers that contribute to plant tolerance to UV-B light. CPD- or 6,4PP-specific photolyases require UV-A/blue light to reverse photodimer formation and restore the original bases (Sancar, 1994). Nucleotide excision repair (NER), an additional DNA repair mechanism, functions without the need for light energy. Several enzymes are involved, resulting in the excision of a small strand of bases flanking, and including, the photodimer. The remaining gap is filled by the normal replication components. *Arabidopsis thaliana* mutants of the photolyases and NER enzymes are hypersensitive when irradiated with UV-B or UV-C, and mutations in the endonucleases involved in NER, especially, seem to have the most dramatic effect on *Arabidopsis* growth (Harlow *et al.*, 1994; Jiang *et al.*, 1997; Landry *et al.*, 1997; Gardner *et al.*, 2009).

Plants have a UV-B-specific signalling pathway that requires *UV RESISTANCE LOCUS 8* (*UVR8*), which has been recently reviewed in detail (Jenkins, 2009; Tilbrook *et al.*, 2013). Dimers of UVR8 function as a UV-B photoreceptor (Rizzini *et al.*, 2011), and the elegant crystallographic and spectroscopic studies of Christie *et al.* (2012) and Wu *et al.* (2012) demonstrated that the absorption of UV-B by specific tryptophan residues in UVR8 causes dissociation of the UVR8 dimer *in vitro*. Subsequent studies showed that the UVR8 monomer is necessary for interaction with CONSTITUTIVELY PHOTOMORPHOGENIC 1 (COP1) and downstream transduction though ELONGATED HYPOCOTYL 5 (HY5) *in planta* (O’Hara and Jenkins, 2012). *uvr8* mutants were originally isolated due to their hypersensitivity to UV-B when grown in the light and lack of chalcone synthase (*CHS*) induction and subsequent accumulation of flavonoids compared with the wild type (wt) (Kliebenstein *et al.*, 2002). However, *uvr8* mutants have also demonstrated lack of hypocotyl growth inhibition in seedlings exposed to UV-B light (Favory *et al.*, 2009; O’Hara and Jenkins, 2012).

Previous work using etiolated *Arabidopsis* seedlings showed that a mutant of the 3′-endonuclease involved in NER, *uvr1-1*, was more sensitive in terms of hypocotyl growth inhibition than the wt after UV-B irradiation (Gardner *et al.*, 2009). The same study reported that a mutant of *UVR8* had similar hypocotyl growth inhibition to the wt after UV-B irradiation. Based on that work, it was hypothesized that UV-B-induced DNA damage, specifically photodimers, leads to hypocotyl growth inhibition in etiolated *Arabidopsis* seedlings. The following experiments show that photomorphogenic inhibition of hypocotyl growth in response to UV-B irradiation in etiolated *Arabidopsis* seedlings is the consequence of cell cycle arrest activated by the accumulation of UV-B-induced DNA photodimers.

## Materials and methods

### Plant material

Seed of the *Arabidopsis* mutant *uvr1-1* (CS8852) was purchased from the Arabidopsis Biological Resource Center (Columbus, OH, USA). *xpf-3*, *xpf sog1-1*, *sog1-1*, and Col:*CYCB1;1-GUS* (Colón-Carmona *et al.* 1999) seeds were generously supplied by A. Britt (UC-Davis, CA, USA). The *uvr8-2* mutant was a gift from G. Jenkins (University of Glasgow, UK). *uvr8-6* was a gift from R. Ulm (University of Geneva, Switzerland). Wt accessions L*er* and Col-0 were purchased from Lehle Seeds (Round Rock, TX, USA).

### Light sources and measurements

UV light sources utilized are as described in Gardner *et al.* (2009). Broad-band UV-B light (FS40-T12-UVB-BP fluorescent tubes, UV Lighting Co., Brook Park, OH, USA) was used for initial fluence response analyses. Monochromatic UV-B light was supplied by a 100W xenon arc lamp through a UV grating monochromator and used for gene expression assays and later fluence–response curves. Fluence rates (μmol m^–2^ s^–1^) for both light sources were measured using a model UVM-SS UV Meter (Apogee Instruments, Logan, UT, USA). Total fluence values (μmol m^–2^) were achieved by varying the time of irradiation. Blue light (BL) for photoreactivation was provided by a Heliospectra L1 prototype light-emitting diode (LED) light source (Heliospectra AB, Göteborg, Sweden) using only the 400nm LEDs. The fluence rate at the level of the plants was ~2.5 μmol m^–2^ s^–1^, measured with an Apogee Model SPEC-UV/PAR spectroradiometer.

### Seed germination and growth

All experiments were conducted with etiolated *Arabidopsis* seedlings. Seeds were germinated and maintained in complete darkness on Whatman #1 filter paper in 60 mm×15mm plastic Petri dishes with 0.5× strength Murashige and Skoog (1962) medium supplemented with 100 μM GA_4_ (Valent Biosciences, North Chicago, IL, USA), herein referred to as MS/GA_4_ solution. Treatments, either UV-B or chemical, were always given shortly after germination when seedlings were ~1–2mm long, ~2–3 d after planting.

### Inhibition of hypocotyl elongation by UV-B

Fluence–response curves for the inhibition of hypocotyl elongation by UV-B were conducted as described in Gardner *et al.* (2009) with minor adjustments. Seeds were germinated as described above with 330 μl of MS/GA_4_ solution. Two- to three-day-old etiolated seedlings were irradiated with either broad-band (10.2 μmol m^–2^ s^–1^) or monochromatic UV-B (290nm, 3.2 μmol m^–2^ s^–1^); the desired fluence was achieved by varying the duration of the radiation. The seedlings were returned to darkness for an additional 2 d and then transferred to a glass plate and digitally photographed. Hypocotyl lengths were measured using ImageJ (http://rsb.info.nih.gov/ij/, last accessed 11 February 2014).

### Photodimer detection

Two- to three-day-old etiolated seedlings (~100–200) were irradiated with 10^4^ μmol m^–2^ monochromatic UV-B at 290nm, frozen in liquid nitrogen immediately after irradiation, and stored at –80 °C. DNA was extracted with a Qiagen DNeasy Plant Mini Extraction kit and all samples were diluted to 0.2ng μl^–1^ with phosphate-buffered saline (pH 7.2). An enzyme-linked immunosorbent assay (ELISA) was performed in a 96-well microtitre plate using monoclonal antibodies specific for either CPDs (TDM-2) or 6,4PPs (64M-2) (MBL International Corporation, Woburn, MA, USA) on 10ng of DNA following the manufacturer’s protocol with additional modifications from Mori *et al.* (1991). CPD and 6,4PP contents were determined by measuring the absorbance at 492nm of six replicates from each DNA sample.

### Hydroxyurea treatment

Dose–response curves for the inhibition of hypocotyl elongation by the radiomimetic agent hydroxyurea (HU; Sigma-Aldrich, St. Louis, MO, USA) were conducted similarly to UV-B fluence–response curves; however, 300 μl of the MS/GA_4_ solution was used for germination. Two- to three-day-old etiolated seedlings were treated with HU over a range of concentrations diluted with 0.5× strength MS (without GA_4_) in a total volume of 100 μl. Two days after treatment, hypocotyls were digitally photographed and measured as described previously. A dish containing seedlings that were not given any additional treatment and one treated with 100 μl of 0.5× strength MS medium were used as controls. The concentration of HU that induced a 50% reduction in hypocotyl elongation was 1mM (Fig. 2A), and this concentration was used for subsequent experiments. When both UV-B and HU were applied, seedlings were first irradiated with UV-B and then given 1mM HU immediately afterward.

### Gene expression

Two- to three-day-old etiolated seedlings (~100–200) were either irradiated with monochromatic UV-B at 290nm, given 1mM HU, or both, and then maintained in the dark until harvest 2–24h after irradiation. Samples were immediately frozen in liquid nitrogen and stored at –80 °C. Total RNA was extracted using a PureLink RNA Mini Kit (Invitrogen) following on-column DNase digestion instructions. Extracts were quantified with a Qubit Fluorometer (Invitrogen) and a Quant-iT BR RNA Assay Kit (Invitrogen). cDNA was synthesized in duplicate from 5ng of total RNA extracts for each reaction using the iScript cDNA Synthesis Kit (Bio-Rad Laboratories). Duplicate reactions were pooled after synthesis and stored at –20 ºC. Real-time reactions were set up in triplicate according to Bio-Rad iQ SYBR Green Supermix instructions and run on the CFX96 Real-Time System (Bio-Rad Laboratories). Gene expression values were automatically calculated by the accompanying CFX Manager 2.0 software using a Livak 2^–ΔΔ*C*T^ method and *ACTIN2* (At3g18780) as the reference gene. Primer sequences used were: *ACTIN2* (At3g18780), *ACTIN* Fwd 5′-GTT GGG ATG AAC CAG AAG GA-3′ and *ACTIN* Rev 5′-GCT CTT CAG GAG CAA TAC GAA G-3′; *CHS* (At5g13930), *CHS* Fwd 5′-CCT GAC ACA TCT GTC GGA GA-3′ and *CHS* Rev 5′-GGT GAG ACC AAC TTC CCT CA-3′; *UDPgtfp* (At1g05680), *UDP* Fwd 5′-CTG GAG TCC TCA GCT TGA CGT A-3′ and *UDP* Rev 5′-TCA CCT TCT GCC TTA ACC CTT A-3′; and *CYCB1;1* (At4g37490), *CYCB1;1* Fwd 5′-CCT CGC AGC TGT GGA ATA TGT-3′ and *CYCB1;1* Rev 5′-TCA ACC ACT CCA CCA GGA TCA-3′.

### CYCB1;1-GUS staining

Two- to three-day-old etiolated seedlings containing a *CYCB1;1-GUS* (β-glucuronidase) reporter construct (Colón-Carmona *et al.*, 1999) were irradiated with broad-band UV-B and harvested immediately (0h) or at various times up to 48h after irradiation. Each time point had a corresponding dark or unirradiated control. During harvest, ~10 seedlings were placed in 5ml of staining solution (100mM disodium phosphate pH 7.0, 1mM X-GlcA, 5% sodium azide) for each sample and incubated at 37 ºC for 2 d. Seedlings were destained with 70% ethanol for 1 d at 65 ºC. GUS expression was visualized using light microscopy. Each experiment was repeated twice and representative seedlings are shown.

## Results

### Nucleotide excision repair mutants are hypersensitive to UV-B

**Fig. 1.**
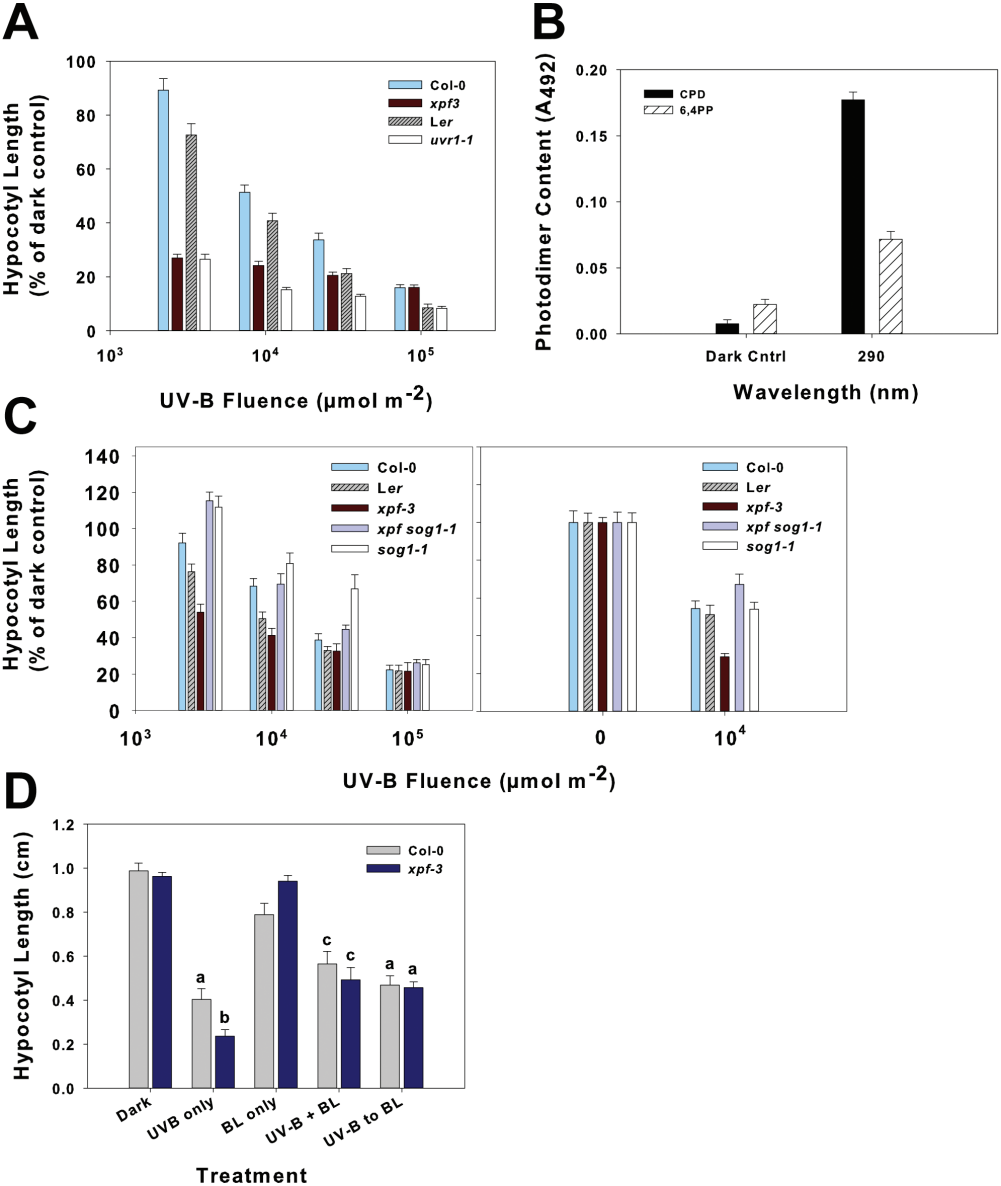
Fluence response for inhibition of hypocotyl growth by UV light in *Arabidopsis* mutants deficient in DNA repair or DNA damage signalling and photodimer content in wild-type Col-0 after UV-B irradiation. (A) Fluence–response curves for nucleotide excision repair (NER) mutants, *xpf-3* (Col-0) and *uvr1-1* (L*er*). Two-day-old etiolated seedlings were irradiated with the total fluence indicated and returned to the dark for an additional 2 d. Data are expressed as a percentage of the unirradiated dark control of the same genotype (±SE). (B) CPD and 6,4PP content in etiolated Col-0 irradiated with 10 000 μmol m^–2^ monochromatic UV-B at 290nm. Content is expressed as mean absorbance at 492nm ±SE (*n*=6). (C) Fluence–response curves for *xpf-3*, *xpf sog1-1* (Col-0/L*er*), and *sog1-1* (Col-0) irradiated with either broad-band (left graph) or narrow-band (right graph) UV-B. Treatment and measurement were as described in (A). (D) Photoreactivation of UV-B-induced hypocotyl growth inhibition in Col-0 and *xpf-3* seedlings. Two-day-old etiolated seedlings were irradiated either with UV-B at 290nm, blue light at 400nm (BL), UV-B at 290nm and BL at 400nm concurrently (UV-B+BL), or UV-B followed by BL irradiation (UV-B, BL), returned to darkness and photographed 2 d later. Total UV-B fluence was 10^4^ μmol m^–2^, and total BL treatment fluence was ~8000 μmol m^–2^ over the same duration as the UV-B irradiation (~52min). Means are displayed ±SE and letters indicate significance (*P*<0.05) based on a Student’s *t*-test between Col-0 wt and *xpf-3* and treatments.

Previously, Gardner *et al.* (2009) tested hypocotyl growth inhibition by UV-B in DNA repair mutants and found that *uvr1-1*, a mutant deficient in the 3′-endonuclease involved in NER, was an order of magnitude more sensitive than the wt (L*er*). Here, hypocotyl growth inhibition by UV-B in *xpf-3*, a mutant deficient in the 5′-endonuclease of NER, was also hypersensitive to UV-B compared with its Col-0 wt, and the inhibition of hypocotyl growth of both NER mutants was greatly increased at the lowest UV-B irradiation treatments (fluences) tested, whereas the wt had only a slight response at those fluences (Fig. 1A). Hypocotyl lengths of the etiolated seedlings are similar before irradiation, and *xpf-3* seedlings are visibly much shorter after 10^4^ μmol m^–2^ UV-B (Supplementary Fig. S1 available at *JXB* online). In the Col-0 wt, the photodimer content of both CPDs and 6,4PPs increased after UV-B irradiation at 290nm compared with the dark control (unirradiated) samples (Fig. 1B). This coincided with the ~40% reduction in hypocotyl growth after the same irradiation treatment (Fig. 1C). Furthermore, BL treatment either before or concurrent with UV-B irradiation reversed the hypersensitivity of *xpf-3* to UV-B irradiation alone (Fig. 1D). This suggests that the increased hypocotyl growth inhibition of *xpf-3* is a photoreactivatable response and a consequence of photodimer accumulation.

### *Suppressor of gamma 1* (*sog1-1*), a γ-irradiation-insensitive mutant, reverses *xpf-3* hypersensitivity to UV-B

Pruess and Britt (2003) reported that after γ-irradiation, *xpf* mutants showed a strong induction of a subset of genes and have delayed growth due to cell cycle arrest in response to an accumulation of double-strand breaks and stalled replication sites. In the same report, they isolated *sog1-1* using a screen for mutations that suppress the γ-irradiation response in *xpf* seedlings (Pruess and Britt, 2003). Therefore, inhibition of hypocotyl elongation of *sog1-1* was measured in response to UV-B and was the same as in the wt (Fig. 1C). In addition, the double mutant *xpf sog1-1* also exhibited a wt response to UV-B, indicating that *sog1-1* reversed hypocotyl growth inhibition by UV-B in *xpf*, which parallels *sog1-1* reversal of γ-irradiation responses in *xpf*. A similar hypocotyl growth reversal was not observed in *xpf* that contains *atm* or *atr* mutations, components involved in DNA damage response signalling (Supplementary Fig. S2 available at *JXB* online). In addition, UV-B hypocotyl growth inhibition was measured in other DNA repair or cell cycle mutants such as *wee1*, and no differences from the wt have been observed (data not shown).

### The radiomimetic compound HU also induces inhibition of hypocotyl growth in *Arabidopsis* seedlings, but *xpf-3* is not hypersensitive to HU

**Fig. 2.**
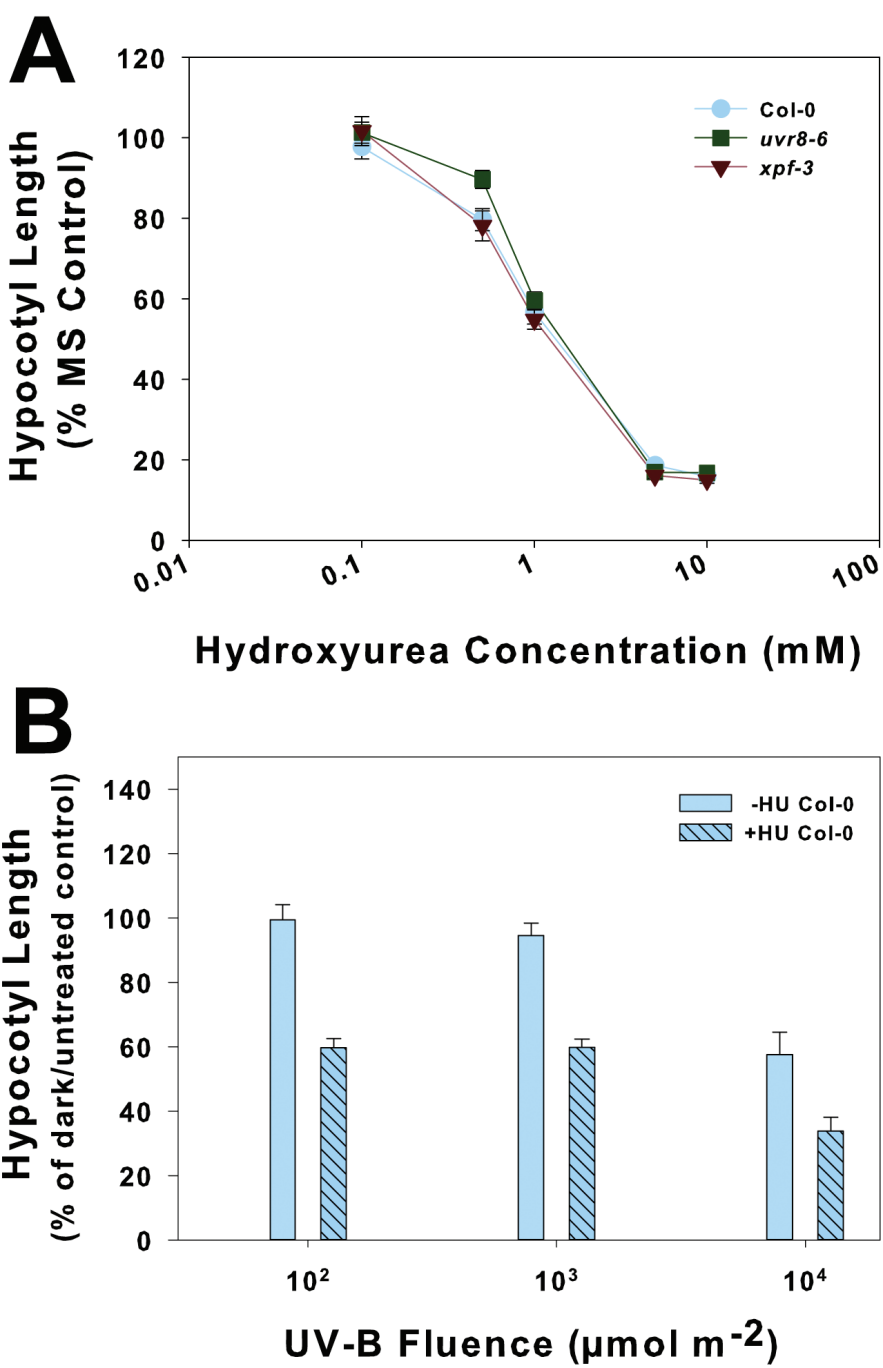
Hypocotyl growth inhibition after hydroxyurea (HU) treatment in *Arabidopsis* seedlings. Two- to three-day-old etiolated seedlings were either (A) treated with HU over a range of concentrations (Col-0 wt, *uvr8-6*, and *xpf-3*) or (B) irradiated with narrow-band UV-B (290nm) with (+HU) or without (–HU) the addition of 1mM HU after irradiation (Col-0 wt only). Seedlings were returned to the dark after treatments for an additional 2 d. Data are expressed as percentage of a 0.5× MS-treated only or untreated dark control (±SE).

Since *xpf* mutants have delayed growth after γ-irradiation by arresting the cell cycle, and SOG1 was required (Pruess and Britt, 2003), it is possible that the hypersensitive hypocotyl growth response to UV-B irradiation in *xpf-3* is due to cell cycle arrest. To determine whether cell cycle arrest affects hypocotyl elongation as UV-B did, HU was applied to etiolated seedlings. HU inhibits DNA replication, resulting in a cell cycle block at the G_1_/S transition (Planchais *et al.*, 2000), and has been used to mimic replication blocks that may result from UV-B- or γ-induced DNA damage (Culligan *et al.*, 2004; Adachi *et al.*, 2011). In etiolated Col-0 wt seedlings HU inhibited hypocotyl elongation in a dose-dependent manner, with a 50% reduction in hypocotyl growth after a 1mM HU application (Fig. 2A). The effect of HU, when given after UV-B irradiation, was not altered after the lower UV-B fluences and was comparable with the hypocotyl growth inhibition after 10^4^ μmol m^–2^ UV-B alone (Fig. 2B). However, there was increased inhibition of hypocotyl growth when HU was applied after 10^4^ μmol m^–2^ UV-B, compared with that same UV-B fluence alone (Fig. 2B), indicating an additive effect of the UV-B irradiation and HU.

**Fig. 3.**
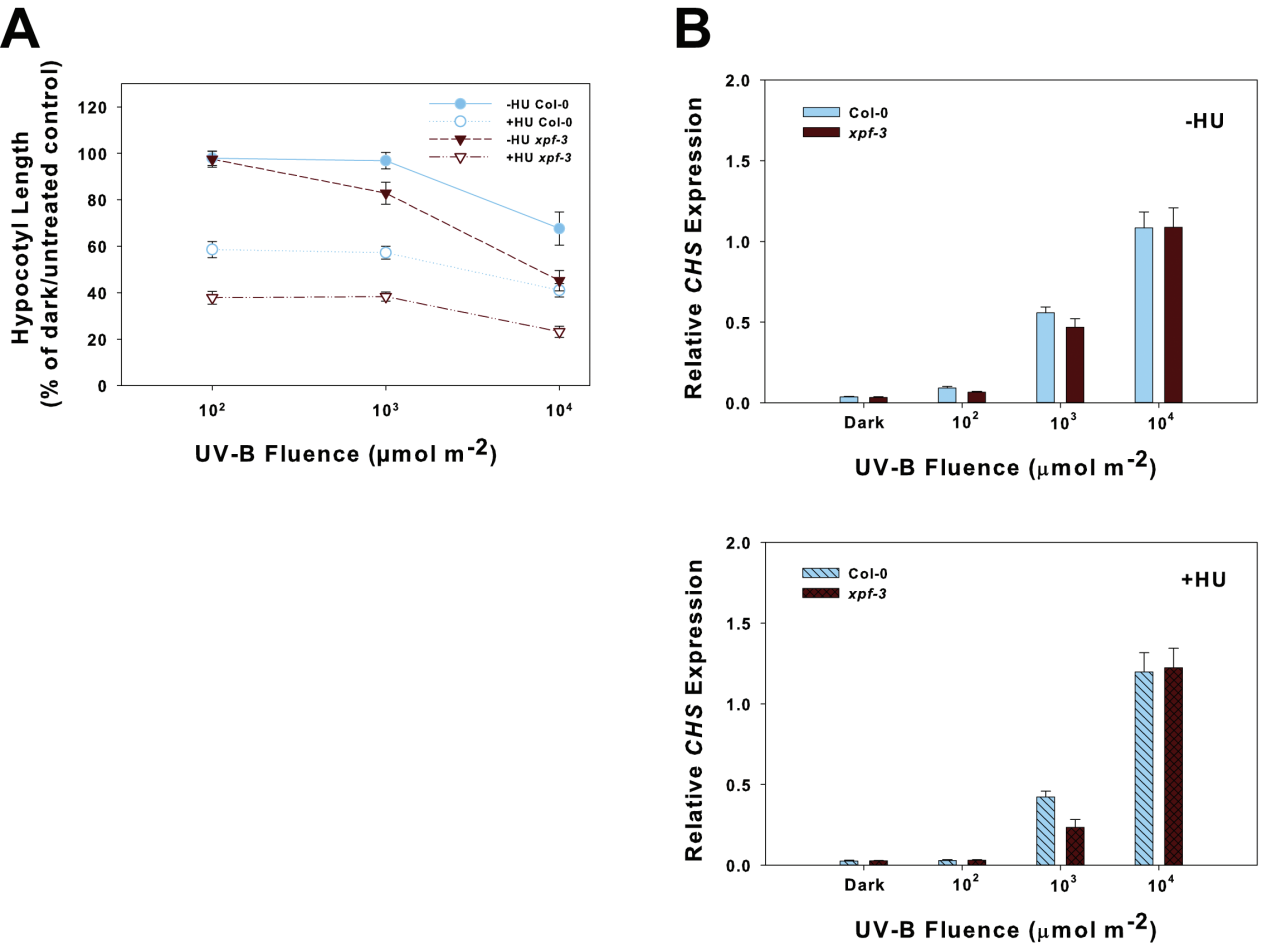
Effect of hydroxyurea (HU) after UV-B irradiation in the nucleotide excision repair (NER) mutant *xpf-3*. (A) Hypocotyl growth inhibition in 2- to 3-day-old etiolated seedlings irradiated with UV-B and subsequently treated with 1mM HU. Circles represent Col-0 wt and triangles represent *xpf-3.* Filled symbols indicate response after UV-B irradiation only (–HU); open symbols indicate response after UV-B irradiation with 1mM HU treatment (+HU). Data are expressed as a percentage of the untreated dark control of the same genotype (±SE). (B) UV-B-specific chalcone synthase (*CHS*) expression in 2- to 3-day-old etiolated seedlings irradiated with UV-B at 290nm. Seedlings were placed back in the dark and harvested 2h later. Expression (±SE; *n*=3) was determined by quantitative real-time PCR using the Livak 2^–ΔΔ*C*T^ method with *ACTIN2* as the reference gene. The top panel shows expression after UV-B irradiation only (–HU). The bottom panel shows expression after UV-B irradiation with 1mM HU treatment (+HU).

Unlike its response to UV-B (Fig. 1A), etiolated *xpf-3* was not hypersensitive to HU treatment alone and showed the same dose–response as the wt (Fig. 2A). HU applied to *xpf-3* after UV-B irradiation had a greater effect on the inhibition of hypocotyl elongation, compared with Col-0 wt (Fig. 3A, open symbols). However, the overall pattern was maintained in both the Col-0 wt and *xpf-3*, in that HU applied after the two lowest UV-B irradiations induced a similar level of hypocotyl growth inhibition, but there was increased growth inhibition when HU was applied after 10^4^ μmol m^–2^ UV-B. The only difference was that *xpf-3* showed an inhibition of hypocotyl elongation after irradiation with 10^3^ μmol m^–2^ UV-B only (without subsequent HU treatment) and the wt did not (Fig. 3A, filled symbols). Therefore, the effects of UV-B and HU appear to be additive, acting independently.

### Nucleotide excision repair is not required for UV-B-specific gene expression of chalcone synthase

Because *xpf-3* is hypersensitive to UV-B in terms of hypocotyl growth inhibition, and as this sensitivity may be due to an accumulation of unrepaired DNA damage, it is possible that other UV-B-specific responses, such as the expression of *CHS*, are also affected. Using monochromatic UV-B at 290nm, *CHS* expression was measured in *xpf-3*. In both Col-0 and *xpf-3*, there was little *CHS* expression in the dark and after 10^2^ μmol m^–2^ UV-B (Fig. 3B). A moderate increase in expression occurred after 10^3^ μmol m^–2^ UV-B and an ~2-fold increase in expression after 10^4^ μmol m^–2^ UV-B. *xpf-3* began to show hypocotyl elongation inhibition after 10^3^ μmol m^–2^ and was strongly inhibited after 10^4^ μmol m^–2^ UV-B (Fig. 3A), but *CHS* expression in *xpf-3* remained similar to that in the wt.

It may be that 10^4^ μmol m^–2^ UV-B irradiation is causing non-specific or general stress responses that include the induction of *CHS* expression (Dixon and Paiva, 1995). If that is the case, then *CHS* expression would have probably been higher in *xpf-3* compared with the wt. Furthermore, adding 1mM HU only to etiolated seedlings did not affect *CHS* expression in either the Col-0 wt or *xpf-3* (Fig. 3B, bottom panel ‘dark’). Finally, *CHS* expression after UV-B irradiation with subsequent HU treatment was similar in the wt and *xpf-3*, as was expression after UV-B alone (Fig. 3B).

### UV-B hypocotyl growth inhibition is distinct from UVR8

**Fig. 4.**
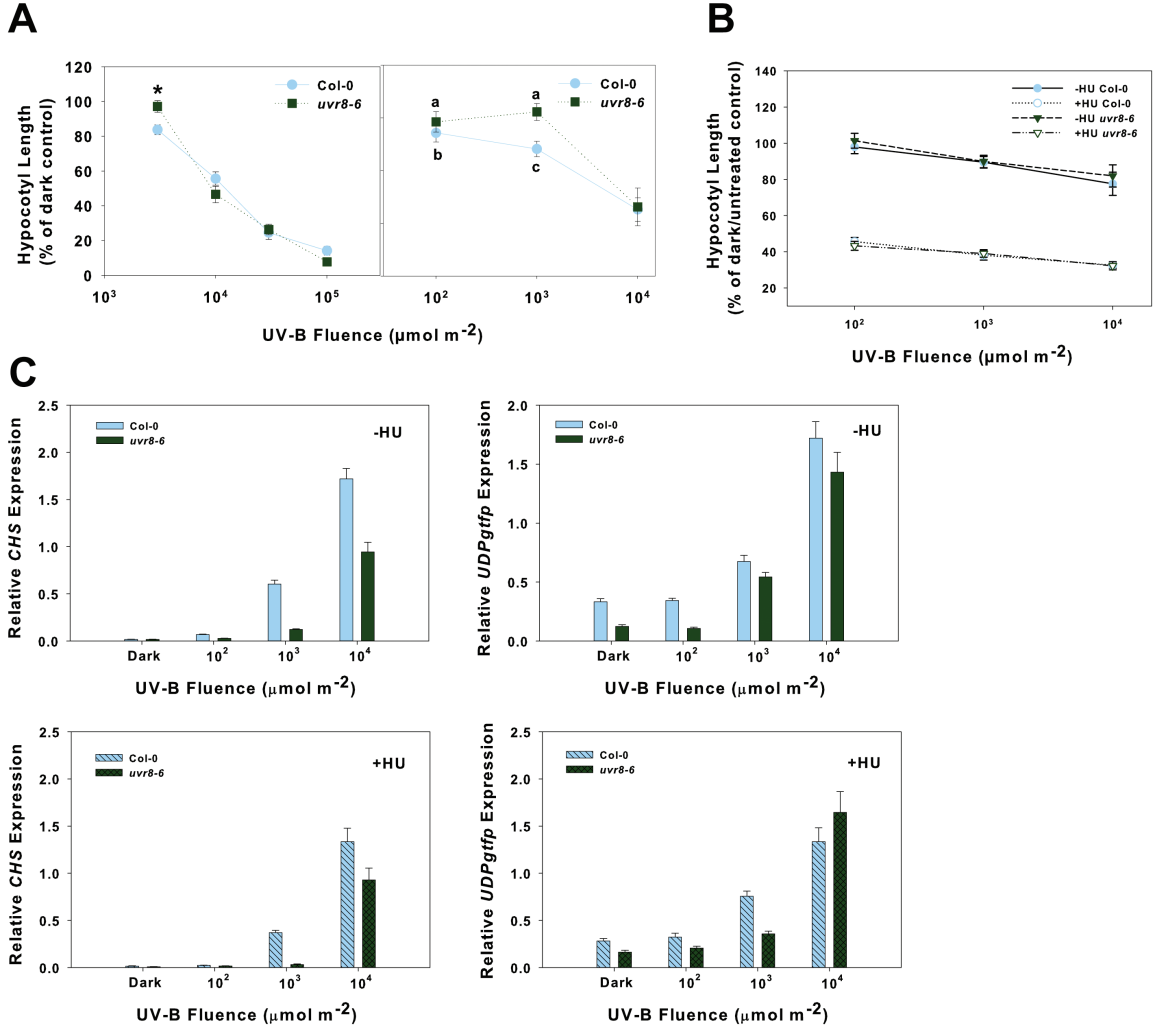
Effect of UV-B irradiation and hydroxyurea (HU) on hypocotyl growth and gene expression in *uvr8-6*. (A) Fluence–response curve for inhibition of hypocotyl growth with either broad-band UV-B (left panel) or narrow-band UV-B at 290nm (right panel). (B) Hypocotyl growth inhibition in etiolated seedlings irradiated with UV-B and subsequently treated with 1mM HU. Circles represent Col-0 wt and triangles represent *uvr8-6.* Filled symbols indicate response after UV-B irradiation only (–HU); open symbols indicate response after UV-B irradiation with 1mM HU treatment (+HU), and lines for Col-0 and *uvr8-6* are superimposable for this response. Growth experiments in (A) and (B) used 2- to 3-day-old etiolated seedlings at the time of treatment. Data are expressed as a percentage of the untreated dark control of the same genotype (±SE); an asterisk (*) indicates a significant difference (*P*<0.05) based on a Student’s *t*-test comparing Col-0 wt and *uvr8-6* at 3×10^3^ μmol m^–2^ UV-B; letters indicate significance (*P*<0.05) based on a Student’s *t*-test between all pair-wise comparisons of Col-0 wt and *uvr8-6* at 10^2^ μmol m^–2^ and 10^3^ μmol m^–2^ UV-B treatments. (C) UV-B-specific gene expression in 2- to 3-day-old etiolated seedlings irradiated with UV-B at 290nm. Seedlings were placed back in the dark and harvested 2h later. Expression (±SE; *n*=3) was determined by quantitative real-time PCR using the Livak 2^–ΔΔ*C*T^ method with *ACTIN2* as the reference gene. The top panels show expression after UV-B irradiation only (–HU). The bottom panels show expression after UV-B irradiation with 1mM HU treatment (+HU). Left panels, *CHS*; right panels, *UDPgtfp*.

*UVR8* encodes a UV-B photoreceptor (Rizzini *et al.*, 2011) responsible for many plant responses to UV-B. However, when etiolated *uvr8-2* mutants were irradiated with UV-B, their hypocotyl growth response was not different from that of the wt (Gardner *et al.*, 2009). Hypocotyl inhibition in response to UV-B in *uvr8-6*, a null mutant (Favory *et al.*, 2009), was also similar to that of the wt after irradiation with both broad-band and monochromatic UV-B (Fig. 4A). Mutants of *COP1* and *HY5* also showed UV-B hypocotyl growth inhibition that was similar to that of the wt (Supplementary Fig. S3 available at *JXB* online). When etiolated *uvr8-6* mutants were irradiated with UV-B, *CHS* expression was not induced until the fluence reached 10^4^ μmol m^–2^ where expression was only about half that of the wt (Fig. 4C). Therefore, while inhibition of hypocotyl elongation in response to UV-B does not require *UVR8* in etiolated seedlings, the induction of *CHS* does. In contrast, *UDPgtfp*, a UV-B-specific gene induced independently of *UVR8* (Brown and Jenkins, 2008), was still induced by UV-B in *uvr8* mutants (Fig. 4C).

When HU was applied to *uvr8-6* either alone or after UV-B irradiation, hypocotyl growth inhibition was not different compared with the wt (Figs 2A, 4B), indicating that the cell cycle response (see below) does not require UVR8. *CHS* expression was not further induced after HU treatment at the lower UV-B fluences. It did have a stronger induction than after 10^4^ μmol m^–2^ UV-B irradiation alone, but it was still lower than the wt (Fig. 4C). The *UVR8*-independent gene, *UDPgtfp*, was also not affected by HU treatment in *uvr8-6* (Fig. 4C). Similar results were seen with *uvr8-2* (Supplementary Fig. S4 available at *JXB* online).

**Fig. 5.**
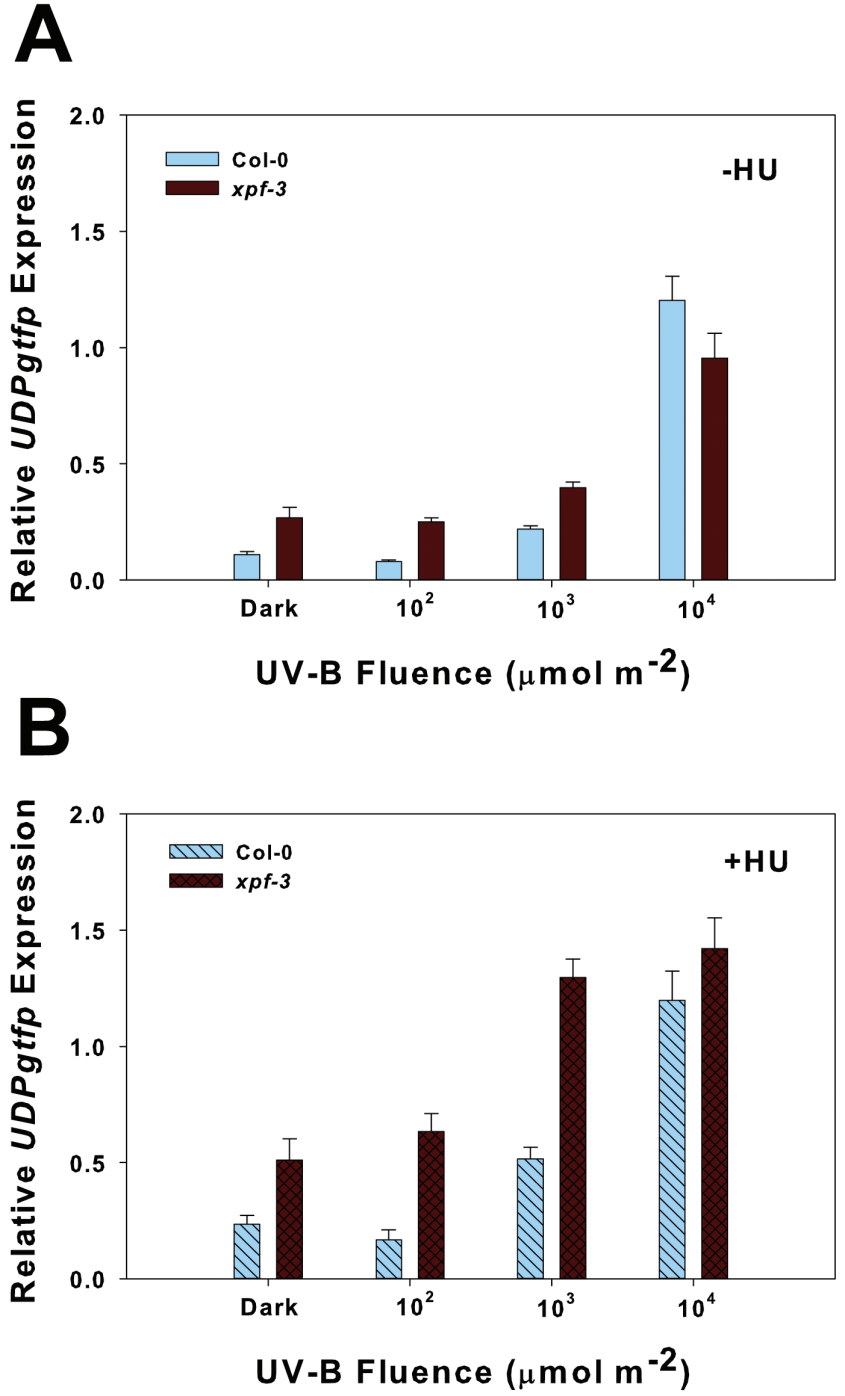
Expression of the UVR8-independent gene *UDPgtfp* in the nucleotide excision repair mutant *xpf-3* and wt after HU treatment. Two- to three-day-old etiolated seedlings were irradiated with UV-B at 290nm with 1mM HU added immediately after irradiation. Seedlings were placed back in the dark and harvested 2h later. Expression (±SE; *n*=3) was determined by quantitative real-time PCR using the Livak 2^–ΔΔ*C*T^ method with *Actin2* as the reference gene. (A) UV-B irradiation only (–HU); (B) UV-B irradiation with 1mM HU treatment (+HU).

In *xpf-3*, *UDPgtfp* expression was strongly induced to a similar degree after 10^4^ μmol m^–2^ UV-B to that in the wt after UV-B irradiation alone (Fig. 5A). However, expression was slightly higher in *xpf-3* after irradiation with the lower UV-B fluences and in the dark (no light treatment). When HU was applied, *UDPgtfp* expression in *xpf-3* was at least 2-fold higher compared with the wt in the dark and at the lowest UV-B fluences tested, but expression was similar after 10^4^ μmol m^–2^ UV-B (Fig. 5B).

### UV-B hypocotyl growth inhibition is caused by cell cycle arrest

**Fig. 6.**
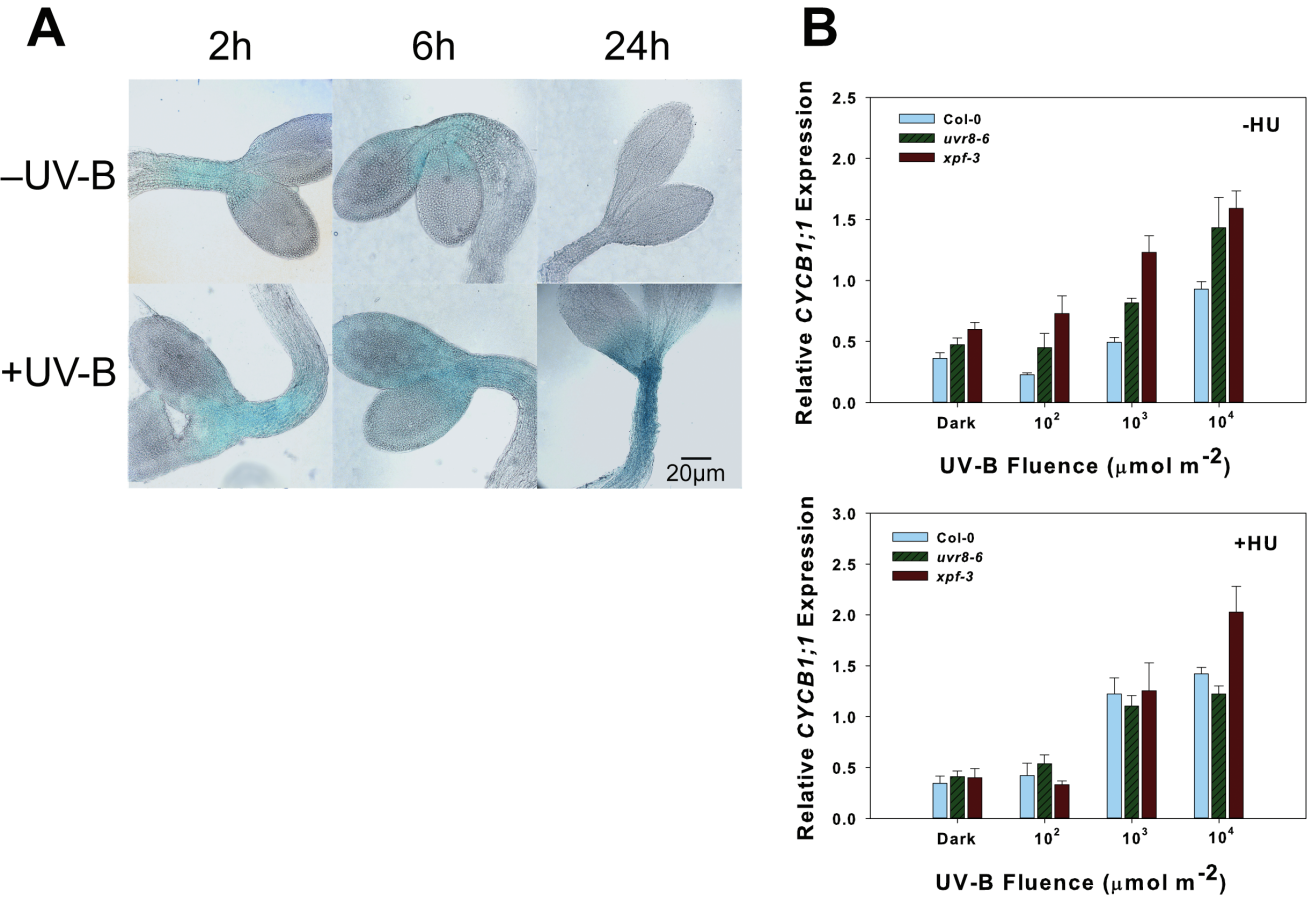
Expression of *CYCB1;1* in etiolated seedlings irradiated with UV-B. (A) CYCB1;1–GUS accumulation in 2- to 3-day-old etiolated Col-0 seedlings irradiated with 10 000 μmol m^–2^ broad-band UV-B. Approximately 10 seedlings were observed from each time point. Photographs show representative samples. (B) *CYCB1;1* expression in Col-0, *uvr8-6*, and *xpf-3* 24h after 100 (10^2^), 1000 (10^3^), or 10,000 (10^4^) μmol m^–2^ UV-B light irradiation at 290nm. ‘Dark’ samples indicate dark/unirradiated control (–HU) and 1mM HU treated/unirradiated control (+HU).

Wt Col-0 seedlings containing a *CYCB1;1-GUS* construct were irradiated with broad-band UV-B, returned to darkness, and harvested 2–48h after irradiation. *CYCB1;1* is a G_2_/M-specific gene that is strongly up-regulated in response to DNA damage from ionizing radiation (Culligan *et al.*, 2006). GUS staining was most prominent at the meristems but also extended into the hypocotyl and cotyledons (Fig. 6A). There was less CYCB1;1–GUS accumulation in dark-grown seedlings overall. Generally, CYCB1;1–GUS accumulation increased over time, peaking ~24h after UV-B irradiation, and this high level of accumulation persisted until at least 48h after irradiation. Interestingly, 48h post-irradiation, staining could be seen along the root and most of the hypocotyl (data not shown).

There was a corresponding induction of *CYCB1;1* expression in Col-0 after 10^4^ μmol m^–2^ UV-B irradiation alone (–HU), but not after the lower UV-B treatments or in the dark (Fig. 6B). This parallels hypocotyl growth inhibition, which was observed after 10^4^ μmol m^–2^ but not after 10^2^ or 10^3^ μmol m^–2^ UV-B in Col-0 (Fig. 2B). Both *uvr8-6* and *xpf-3* had higher expression of *CYCB1;1* than in the wt after UV-B irradiation alone at each fluence (Fig. 6B). Expression of *CYCB1;1* was highest in *xpf-3*, which parallels its hypocotyl growth response to UV-B (Fig. 3A). However, the higher expression in *uvr8-6* than in the wt after each UV-B irradiation (Fig. 6B, –HU) is in contrast to its hypocotyl response after UV-B irradiation (Fig. 4A, B). *CYCB1;1* expression was not induced in the dark by HU treatment alone in either Col-0, *uvr8-6*, or *xpf-3* (Fig. 6B, +HU). The expression remained similar among all three genotypes when HU was applied after UV-B irradiation, except after 10^4^ μmol m^–2^ UV-B, where *xpf-3* showed the highest expression of *CYCB1;1* (Fig. 6B, +HU).

## Discussion

### UV-B inhibition of hypocotyl growth is a consequence of cell cycle arrest initiated by photodimer formation

Specific photomorphogenic responses to UV-B include hypocotyl growth inhibition (Kim *et al.*, 1998; Shinkle *et al.*, 2004), changes in gene expression (Ulm *et al.*, 2004; Brown *et al.*, 2005), and cotyledon expansion (Kim *et al.*, 1998), among others (Barnes *et al.*, 2005; Gerhardt *et al.*, 2005; Ulm, 2006). UVR8 is required, along with the transcription factor HY5, for UV-B-specific induction of *CHS* (Ulm *et al.*, 2004; Brown *et al.*, 2005; Brown and Jenkins, 2008). CHS catalyses the biosynthesis of flavonoids, which is an important element of UV-B light tolerance in plants (Favory *et al.*, 2009; Gruber *et al.*, 2010). Responses to DNA damage caused by UV-B light are often not considered photomorphogenic, but rather non-specific, stress-like responses that are also induced by other stimuli (Boccalandro *et al.*, 2001; Brosché and Strid, 2003). However, the formation of photodimers is specific to UV-B light. Here, evidence is provided that the inhibition of hypocotyl growth in response to UV-B irradiation in etiolated *Arabidopsis* is a consequence of cell cycle arrest that is initiated by photodimer formation.

The inhibition of hypocotyl elongation is a classic photomorphogenic response, and the present results with the *xpf-3* mutant (Fig. 1A, C) indicate that DNA damage, specifically the accumulation of unrepaired photodimers (Fig. 1B), influences this response after UV-B irradiation. The hypersensitivity of *xpf-3* to UV-B irradiation may not be surprising; however, these seedlings are completely viable and can be transferred to soil and grown to seed despite the severe inhibition of growth (Gardner *et al.*, 2009). In etiolated wt *Arabidopsis*, with functional XPF, there may still be some DNA damage, but the plant is able to maintain cellular processes without growth consequences. However, at higher UV-B fluences, ≥30 000 μmol m^–2^, DNA damage probably accumulates in the wt to a level where seedlings are unable to sustain timely DNA repair, and the hypocotyl growth response approaches that of the NER mutants (Fig. 1A). Therefore, *xpf-3* seedlings may sustain an increased accumulation of photodimers after UV-B irradiation, due to their inability to repair DNA damage, but are in a state of arrested growth until the excess damage is repaired.

XPF is a 5′-endonuclease that mainly functions in NER in plants, but it can also function in mitotic recombination and repair of double-strand breaks (Bardwell *et al.*, 1994; Gallego *et al.*, 2000). In addition, it probably has some role in the DNA damage signalling network regulated by the protein kinases ATAXIA-TELANGIECTASIA MUTATED (ATM) and ATM AND RAD3-RELATED (ATR) that recognize double-strand breaks and replication blocks, respectively (Garcia *et al.*, 2003; Culligan *et al.*, 2004). Downstream transduction from both ATM and ATR occurs through SOG1, a transcription factor responsible for the expression of several genes induced after γ-irradiation (Yoshiyama *et al.*, 2009). The delayed growth and inhibited transcriptional response to γ-irradiation in *xpf* mutants is reversed in the absence of SOG1 (Pruess and Britt, 2003).

A distinct signalling mechanism for γ-radiation in plants is unlikely due to the almost non-existent levels of γ-radiation experienced on earth. Thus, it seems logical that this signalling pathway would function to maintain genome integrity primarily in response to UV-B irradiation. SOG1 does appear to function in responses to UV-B-induced DNA damage since the *sog1-1* mutation reversed the UV-B-hypersensitive phenotype of *xpf* (Fig. 1C). This reversal indicates a loss of signal transduction through SOG1 that is initiated either directly from UV-B-specific photodimers or from stalled replication or transcription sites due to photodimer accumulation, a typical result of UV-B light absorption by DNA (Culligan *et al.*, 2004; Curtis and Hays, 2007), rather than double-strand breaks. This possible UV-B signalling through SOG1 appears to be independent of ATM and ATR (Supplementary Fig. S2 available at *JXB* online).

Cell cycle arrest is the ultimate consequence of signalling through SOG1, and it may be responsible for inhibiting the growth of etiolated seedlings after UV-B irradiation. In wt *Arabidopsis* containing a *CYCB1;1-GUS* reporter construct, expression was low in dark-grown seedlings and much higher after UV-B irradiation (Fig. 6A). The accumulation of CYCB1;1–GUS that was sustained until ~48h after UV-B irradiation is consistent with the time course of hypocotyl elongation inhibition reported by Gardner *et al.* (2009), who showed that hypocotyl growth was inhibited within 6h after UV irradiation and lasted until 3–4 d later.

The alteration of cell cycle progression is a known consequence of UV-B light irradiation. Root growth in *atr* mutants is hypersensitive to replication-blocking agents, including UV-B light, due to a loss in regulation of a G_2_-phase cell cycle checkpoint (Culligan *et al.*, 2004). *Arabidopsis* mutants more tolerant to UV-B underwent extra rounds of endoreduplication in hypocotyl cells (Hase *et al.*, 2006) and were later shown to lack an inhibitor of a complex that promotes cell division (Heyman *et al.*, 2011). Both cell division and elongation contribute to overall growth (Inzé and De Veylder, 2006). In hypocotyls, the bulk of growth is due to cell elongation, with cells that undergo multiple rounds of endoreduplication in the light as well as the dark (Gendreau *et al.*, 1997). A cell cycle block, especially one that inhibits DNA replication such as UV-B light or HU, could conceivably affect elongation and division. Endoreduplication may, in part, be a trigger for cell expansion and elongation (Melaragno *et al.*, 1993). Therefore, if endoreduplication is inhibited, elongation may be as well. Cell division is required initially to supply the elongating cells (Gendreau *et al.*, 1997), and a disruption in DNA replication could also inhibit this, contributing to an overall inhibition of growth in the hypocotyl after UV-B irradiation.

The photoreactivation experiment shown in Fig. 1D provides further evidence that the inhibition of hypocotyl growth in etiolated seedlings is a consequence of photodimer formation. Based on the report of Hada *et al.* (2000) that the action spectrum of higher plant CPD photolyases has a maximum of 400nm, seedlings were treated with 400nm BL either during or immediately following the UV-B treatment. While x*pf-3* showed hypersensitivity to UV-B alone, as expected, BL reversed the mutant phenotype. This suggests that photoreactivation rapidly repairs the photodimers that cannot be repaired by NER in *xpf-3*, and additional inhibition of elongation does not occur.

### Inhibition of hypocotyl growth by UV-B is distinct from that caused by HU

To indicate further that a cell cycle block can result in a similar growth phenotype to UV-B, HU was used to simulate the effects of UV-B irradiation on hypocotyl growth inhibition. HU inhibits DNA replication and induces a G_1_ cell cycle block (Planchais *et al.*, 2000), and etiolated seedlings treated with HU showed an inhibition of hypocotyl elongation in a dose-dependent manner (Fig. 2A). Although hypocotyl growth was inhibited in etiolated *Arabidopsis* seedlings by both UV-B and HU, their effects appear to be independent. *xpf-3* showed hypersensitivity to UV-B (Fig. 1), but had the same response to HU as the wt (Fig. 2A), further suggesting that photodimers may ultimately be responsible. The independent effects of UV-B light and HU on hypocotyl growth inhibition are also clear in that UV-B results in the accumulation of *CYCB1;1*, while HU treatment in the dark does not (Fig. 6B). This emphasizes that there may be multiple mechanisms by which hypocotyl growth can be inhibited, since *CYCB1;1* is required at the G_2_/M transition and HU blocks the cell cycle at the G_1_/S transition.

UV-B-specific expression of *CHS* and the lack of increased expression in response to HU were similar in xpf-3 compared to the wt (Fig. 3B). Since the *xpf-3* mutant and the wt both have intact UVR8, UV-B-specific *CHS* expression would not be expected to be different from that of the wt unless photodimer formation had some effect on *CHS* expression. This also showed that the UV-B irradiation and HU treatment themselves did not simply induce a general stress response in *xpf-3* that resulted in increased *CHS* expression (Dixon and Paiva, 1995).

### Inhibition of hypocotyl growth of etiolated seedlings by UV-B is largely independent of UVR8

The UV-B-specific hypocotyl growth inhibition that was observed in etiolated seedlings is a photomorphogenic response that occurs largely independently of the UVR8 photoreceptor (Fig. 4A). There has been at least one report of two distinct UV-B photomorphogenic pathways, where DNA was implicated as the chromophore in one of them (Shinkle *et al.*, 2004). UV-B-induced signalling pathways that are independent of UVR8 have also been reported (Brown and Jenkins, 2008; Wargent *et al.*, 2009; González Besteiro *et al.*, 2011) and further indicate that other UV-B perception mechanisms are present in plants. Brown and Jenkins (2008) described a high-fluence rate response that probably overlaps with oxidative stress or wound signalling pathways that induced gene expression specifically in response to UV-B irradiation, but did not require *UVR8*. UVR8 was shown to be necessary for normal leaf development and expansion in response to UV-B irradiation through regulation of endoreduplication and stomatal differentiation, but reduced cell divisions in the leaf epidermis were not dependent on UVR8 (Wargent *et al.*, 2009). Reactive oxygen species (ROS) signalling pathways, such as those mediated by mitogen-activated protein kinases (MAPKs), are activated by UV-B irradiation (Holley *et al.*, 2003; Kalbina and Strid, 2006). The MKP1 pathway, specifically, functions independently of *UVR8* (González Besteiro *et al.*, 2011). Oxidative stress can be an accompanying problem when irradiating green, photosynthetic tissue with UV-B light due to a disruption of electron transport through photosystem II (Jansen *et al.*, 1996; Vass *et al.*, 1996), and may explain the necrotic phenotype of plants that lack flavonoid production such as *uvr8* (Gruber *et al.*, 2010; González Besteiro *et al.*, 2011).

*UDPgtfp* was one of the *UVR8*-independent, UV-B-specific genes previously reported (Brown and Jenkins, 2008). This particular UDP-glucosyltransferase is rapidly induced by H_2_O_2_ and glycosylates the auxin indole-3-butyric acid (IBA) to regulate growth and physiological responses to biotic and abiotic stress (Tognetti *et al.*, 2010). The present results confirmed its UV-B-specific induction independent of *UVR8* (Fig. 4C). The interplay of ROS formation and signalling with UV-B responses was not directly tested here. However, because etiolated tissue was used in these experiments, ROS formation, at least resulting from disrupted photosynthesis, should be minimal. The higher expression of *UDPgtfp* in the *xpf-3* mutant (Fig. 5) may reveal a novel function of this gene in the DNA damage response from blocked replication that leads to cell cycle arrest, although expression due to ROS formation and signalling cannot be ruled out.

### Inhibition of hypocotyl elongation by UV-B via cell cycle arrest is a property of etiolated seedlings

It is reported here that *uvr8* shows inhibition of hypocotyl growth by UV-B that is similar to the wt (Fig. 4; Supplementary Figs S1, S4 at *JXB* online), which is an apparent contradiction to previously documented *uvr8* phenotypes. It is important to distinguish that the growth conditions used here of complete darkness with pulses of UV-B light are quite different from those of other studies that showed that *uvr8* mutants grown under continuous white light conditions, either with or without supplementary UV-B light, lacked the UV-B-induced hypocotyl growth inhibition of the wt (Favory *et al.*, 2009). Also, overexpression of UVR8 resulted in hyperinduction of *CHS* along with increased hypocotyl growth inhibition by UV-B light (Favory *et al.*, 2009), where here the hypersensitive UV-B hypocotyl growth observed in *xpf-3* was not accompanied by enhanced *CHS* induction (Fig. 3).

As noted in initial studies (Gardner *et al.*, 2009), it was decided to use completely etiolated plants in order to reduce the possibility of detecting events that are induced by other, non-UV-related, photoreceptors and to eliminate complicating factors that might be associated with de-etiolation, such as the production of chlorophyll and other screening pigments, or the synthesis of the photosynthetic apparatus. Therefore, it is difficult to compare the fluence–response sensitivity reported here directly with that reported by others. For example, Favory *et al.* (2009) measured growth inhibition in light-grown plants after 4 d of continuous UV-B treatment at 1.5 μmol m^–2^ s^–1^, corresponding to a total fluence of ~5×10^5^ μmol m^–2^. They also reported experiments with 1h or 6h of UV-B at 1.5 μmol m^–2^ s^–1^, resulting in 5.4×10^3^ μmol m^–2^ and 3.24×10^4^ μmol m^–2^ total UV-B. This is on the same order of the experiments reported here at 10^4^ μmol m^–2^, which was given over 16min for the broad-band source or over 52min at 290nm with the monochromator.

The present results are also different from the original isolation of *uvr8* that reported it to be more sensitive to UV-B irradiation than the wt (Kliebenstein *et al.*, 2002). *uvr8* sensitivity is more pronounced in plants that have had an ‘acclimation’ period to low levels of UV-B supplied with continuous white light (González Besteiro *et al.*, 2011) and is consistent with the lack of *CHS* expression in *uvr8* mutants (Kliebenstein *et al.*, 2002; Brown and Jenkins, 2008; Favory *et al.*, 2009). Therefore, a sensitive phenotype in light-grown *uvr8* plants may be a result of damage due to a lack of flavonoids to screen the UV-B. Likewise, the measurements in etiolated wt seedlings presented here are taken before a protective effect from the induction of flavonoid biosynthesis can be observed (Supplementary Fig. S1 at *JXB* online). *CHS* expression in the etiolated *uvr8* mutants (Fig. 4C; Supplementary Fig. S4), however, is consistent with previous reports, regardless of growth conditions (Kliebenstein *et al.*, 2002; Brown and Jenkins, 2008; Favory *et al.*, 2009).

Another possible explanation for the UV-B inhibition seen in the wt but not in *uvr8* by others (Favory *et al.*, 2009) may be due to the increase in flavonoids induced by UV-B. It has long been known that flavonoids can inhibit auxin transport (Stenlid, 1976; Jacobs and Rubery, 1988; Gardner and Sanborn, 1989), and this inhibition of auxin transport could result in inhibition of hypocotyl elongation in the wt. In *uvr8*, flavonoid accumulation would not occur in response to UV-B, and auxin transport and growth would not be inhibited. A similar explanation may apply to the slight hyposensitivity that is sometimes observed in *uvr8* at low fluences of UV-B (Fig. 4A). At 10^3^ μmol m^–2^ UV-B, there is only slight inhibition of growth, whereas the same fluence causes a substantial increase in *CHS* expression in the wt (Fig. 4C). Perhaps the CHS-derived flavonoids in the wt cause a slight inhibition of growth at very low fluences, and these would be absent in *uvr8*. Testing this hypothesis on the relative contribution of flavonoids and auxin transport is beyond the scope of the present study but will be the subject of future investigation.

In conclusion, the results presented here show that there is an underlying pathway specific to plant responses to UV-B, distinct from signal transduction through *UVR8*, that influences early *Arabidopsis* seedling growth shortly after germination. This pathway appears to originate from UV-B-induced DNA photodimers and results in photomorphogenic inhibition of hypocotyl growth through a disruption in the cell cycle.

## Supplementary data

Supplementary data are available at *JXB* online.

Figure S1. Response of etiolated *Arabidopsis* seedlings to monochromatic UV-B irradiation.

Figure S2. UV-B fluence response of hypocotyl growth inhibition in DNA damage response mutants.

Figure S3. UV-B fluence response of hypocotyl growth inhibition in *hy5* and *cop-1*.

Figure S4. Effect of UV-B irradiation and hydroxyurea (HU) on hypocotyl growth and gene expression in *uvr8-2*.

Supplementary Data
