# Supplementary material for: UV-B inhibition of hypocotyl growth in etiolated Arabidopsis thaliana seedlings is a consequence of cell cycle arrest initiated by photodimer accumulation
Source: J Exp Bot. 2014 Mar 3;65(11):2949–61. doi: 10.1093/jxb/eru035 (PMC4056539; doi:10.1093/jxb/eru035)
Supplement: Supplementary Data [file supp_65_11_2949__index.html]

UV-B inhibition of hypocotyl growth in etiolated Arabidopsis thaliana seedlings is a consequence of cell cycle arrest initiated by photodimer accumulation — UV-B inhibition of hypocotyl growth in etiolated Arabidopsis thaliana seedlings is a consequence of cell cycle arrest initiated by photodimer accumulation — Supplementary Data 

# UV-B inhibition of hypocotyl growth in etiolated *Arabidopsis thaliana* seedlings is a consequence of cell cycle arrest initiated by photodimer accumulation

## Supplementary Data

Data files

**Files in this Data Supplement:**

- Supplementary Data - Supplementary Data
