# Supplementary material for: UV-B inhibition of hypocotyl growth in etiolated Arabidopsis thaliana seedlings is a consequence of cell cycle arrest initiated by photodimer accumulation
Source: J Exp Bot. 2014 Mar 3;65(11):2949–61. doi: 10.1093/jxb/eru035 (PMC4056539; doi:10.1093/jxb/eru035)
Supplement: Supplementary Data [file supp_eru035_jexbot110650_file001.pdf]

Journal of Experimental Botany

UV-B inhibition of hypocotyl growth in etiolated *Arabidopsis thaliana* seedlings is a consequence of cell-cycle arrest initiated by photodimer accumulation

Jessica J. Biever, Doug Brinkman, and Gary Gardner

Supplementary material:

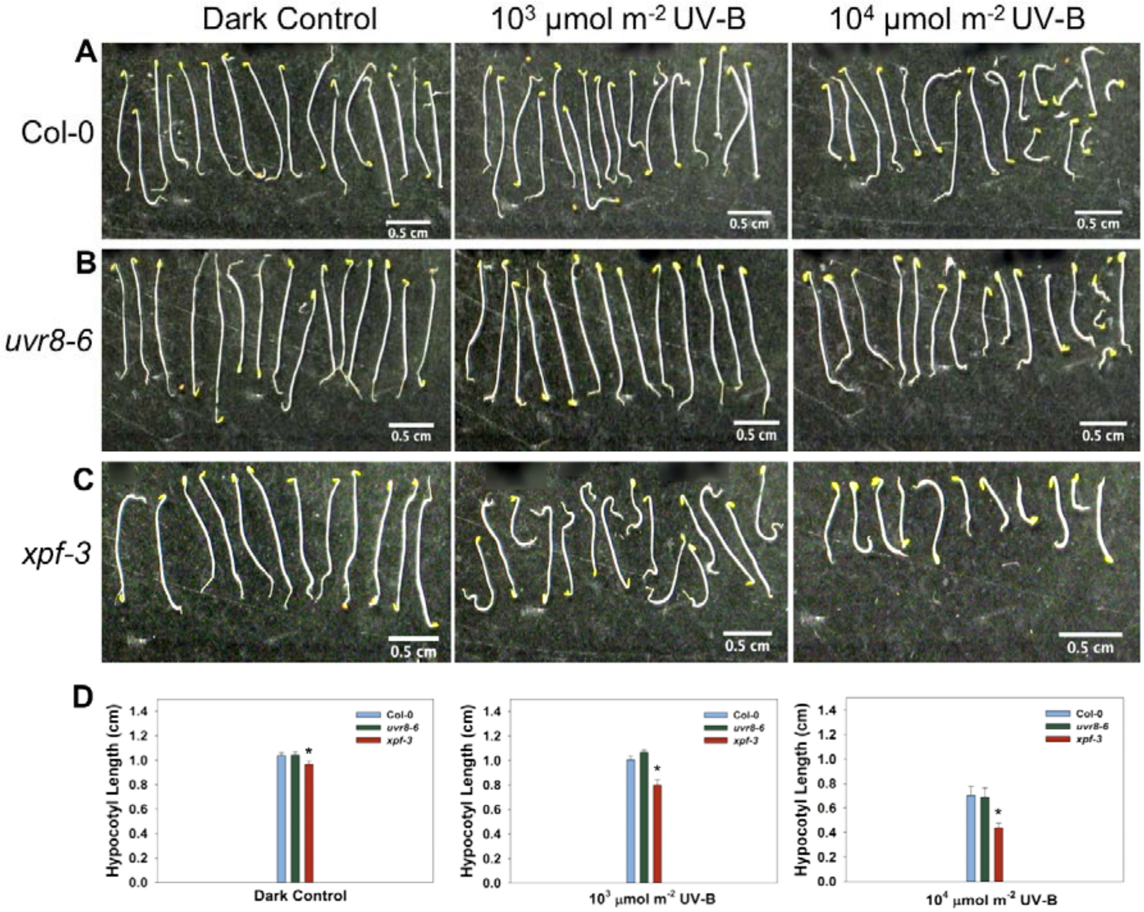

Fig. S1. Response of etiolated Arabidopsis seedlings to monochromatic UV-B irradiation. **A-C)** Hypocotyl growth inhibition in two- to three-day-old etiolated seedlings irradiated with narrow band UV-B at 290 nm. Seedlings were returned to darkness after UV-B irradiation and photographed two days later. **D)** Mean hypocotyl lengths (cm) of the seedlings shown for each treatment (± S.E.). Asterisks (*) denote significance ($p<0.05$) based on the Student's t-test comparison between Col-0 wt and each mutant.

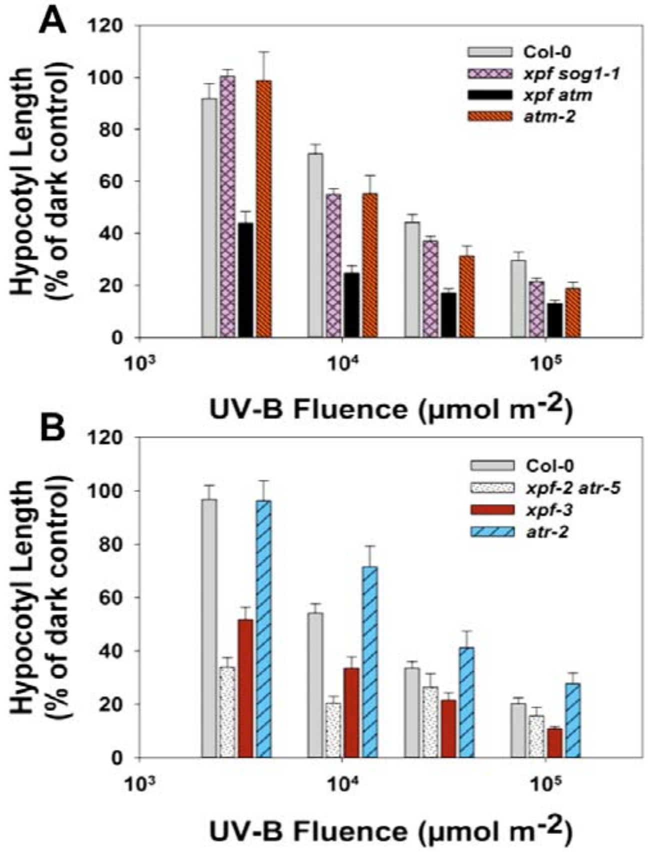

Fig. S2. UV-B fluence response of hypocotyl growth inhibition in DNA damage response mutants. Two- to three-day-old etiolated seedlings were irradiated with broad band UV-B. Seedlings were returned to darkness after UV-B irradiation and photographed two days later. Data are expressed as percent of the untreated dark control of the same genotype (± S.E.). **A)** *xpf sog1-1*, *xpf atm,* and *atm* (SALK_040423C) Arabidopsis mutants. *xpf atm* displays a *xpf-3* hypersensitive UV-B phenotype regarding hypocotyl growth inhibition and is unlike *xpf sog1-1* and the single *atm* mutant that are similar to wt. **B)** *xpf-2 atr-5*, *xpf-3*, and *atr-2* (SALK_032841C) Arabidopsis mutants. *xpf-2 atr-5* mutant also has a *xpf-3* hypersensitive UV-B phenotype regarding hypocotyl growth inhibition, where *atr-5* is similar to wt. Double mutants *xpf atm* and *xpf-2 atr-5* were provided courtesy of Dr. Anne Britt (UC-Davis, CA, USA).

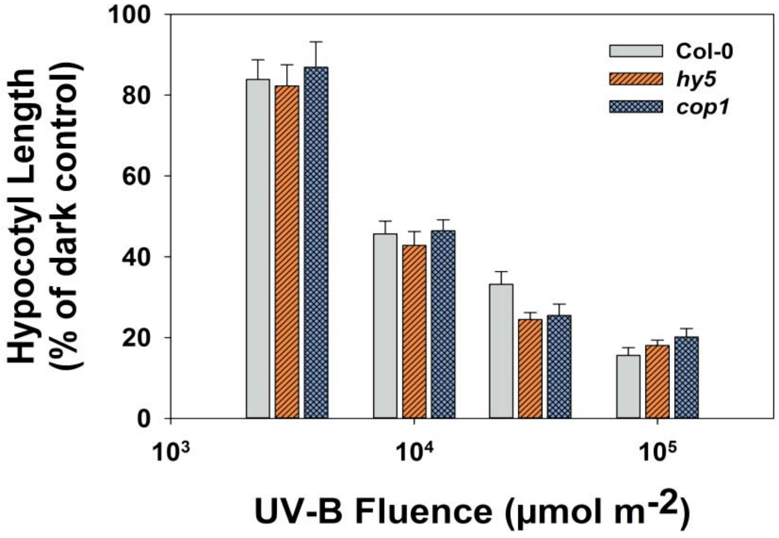

Fig. S3. UV-B fluence response of hypocotyl growth inhibition in *hy5* and *cop1*. Two- to three-day-old etiolated seedlings were irradiated with broad band UV-B. Seedlings were returned to darkness after UV-B irradiation and photographed two days later. Data are expressed as percent of the untreated dark control of the same genotype (± S.E.). HY5 and COP1 are components in the UVR8 photoreceptor signaling pathway in response to UV-B. Mutants of *hy5* and *cop1* have similar hypocotyl growth inhibition as wt after UV-B irradiation.

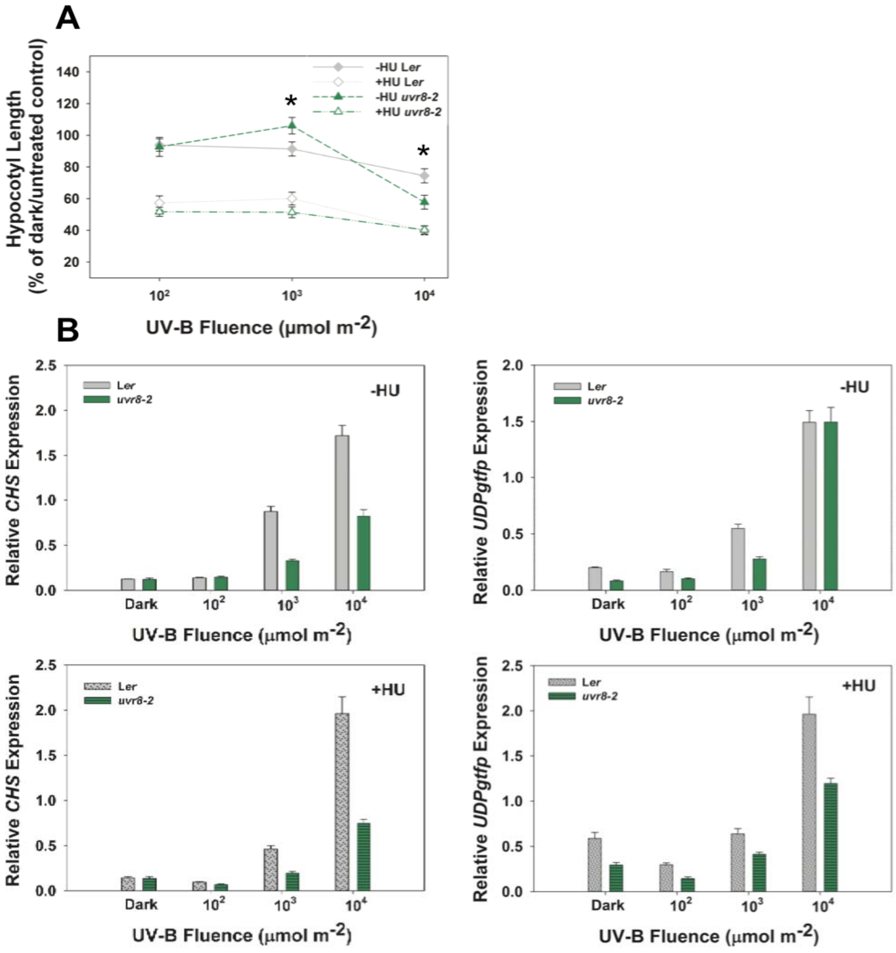

Fig. S4. Effect of UV-B irradiation and hydroxyurea (HU) on hypocotyl growth and gene expression in *uvr8-2*. **A)** Hypocotyl growth inhibition in two- to three-day-old etiolated seedlings irradiated with narrow band UV-B at 290 nm and subsequently treated with 1 mM HU. Circles represent L*er* wt and triangles represent *uvr8-2*. Filled symbols indicate response after UV-B irradiation only (-HU); open symbols indicate response after UV-B irradiation with 1 mM HU treatment (+HU). Data are expressed as percent of the untreated dark control of the same genotype (± S.E.); asterisks (*) indicate significance ($p<0.05$) based on the Student's t-test between L*er* wt and *uvr8-2* at each fluence (in the absence of HU). **B)** UV-B -specific gene expression in two- to three-day-old etiolated seedlings irradiated with UV-B at 290 nm. Seedlings were placed back in the dark and harvested 2 h later. Expression (± SE; n= 3) was determined by quantitative real-time PCR using the Livak $2^{-\Delta\Delta CT}$ method with *ACTIN2* as the reference gene. Top panels show expression after UV-B irradiation only (-HU). Bottom panels show expression after UV-B irradiation with 1 mM HU treatment (+HU). Left panels: *CHS*; right panels: *UDPgtfp*.
